# The relationship between mood and sleep in different female reproductive states

**DOI:** 10.1186/1471-244X-14-177

**Published:** 2014-06-16

**Authors:** Elena Toffol, Nea Kalleinen, Anna Sofia Urrila, Sari-Leena Himanen, Tarja Porkka-Heiskanen, Timo Partonen, Päivi Polo-Kantola

**Affiliations:** 1 Department of Mental Health and Substance Abuse Services, National Institute for Health and Welfare (THL), Mannerheimintie 170, P.O. Box 30, Helsinki FI-00271, Finland; 2 Department of Physiology, Sleep Research Unit, University of Turku, Turku, Finland; 3 Heart Center, Turku University Hospital and University of Turku, Turku, Finland; 4 Department of Physiology, University of Helsinki, Helsinki, Finland; 5 Department of Adolescent Psychiatry, Helsinki University Central Hospital, Helsinki, Finland; 6 Department of Clinical Neurophysiology, Pirkanmaa Hospital District, Tampere, Finland; 7 Faculty of Medicine, University of Tampere, Tampere, Finland; 8 Department of Obstetrics and Gynecology, Turku University Hospital and University of Turku, Turku, Finland

**Keywords:** Perimenopause, Postmenopause, Reproduction, Sleep stage

## Abstract

**Background:**

Sleep is disrupted in depressed subjects, but it also deteriorates with age and possibly with the transition to menopause. The nature of interaction between mood, sleep, age and reproductive state is not well-defined. The aim of this study was to evaluate the relationship between mood and sleep among healthy women in different reproductive states.

**Methods:**

We analyzed data from 11 younger (20–26 years), 21 perimenopausal (43–51 years) and 29 postmenopausal (58–71 years) healthy women who participated in a study on menopause, sleep and cognition. The 21-item Beck Depression Inventory (BDI) was administered to assess mood. Subjective sleep quality was assessed with the Basic Nordic Sleep Questionnaire (BNSQ). Objective sleep was measured with all-night polysomnography (PSG) recordings. Perimenopausal and younger women were examined during the first days of their menstrual cycle at the follicular phase.

**Results:**

Among younger women, less arousals associated with higher BDI total scores (*p* = 0.026), and higher SWS percentages with more dissatisfaction (*p* = 0.001) and depressive-somatic symptoms (*p* = 0.025), but with less depressive-emotional symptoms (*p* = 0.001). In specific, less awakenings either from REM sleep or SWS, respectively, associated with more punishment (*p* = 0.005; *p* = 0.036), more dissatisfaction (*p* < 0.001; *p* = 0.001) and more depressive-somatic symptoms (*p* = 0.001; *p* = 0.009), but with less depressive-emotional symptoms (*p* = 0.002; *p* = 0.003). In perimenopausal women, higher BNSQ insomnia scores (*p* = 0.005), lower sleep efficiencies (*p* = 0.022) and shorter total sleep times (*p* = 0.024) associated with higher BDI scores, longer sleep latencies with more depressive-somatic symptoms (*p* = 0.032) and longer REM latencies with more dissatisfaction (*p* = 0.017). In postmenopausal women, higher REM percentages associated with higher BDI total scores (*p* = 0.019) and more depressive-somatic symptoms (*p* = 0.005), and longer SWS latencies with more depressive-somatic symptoms (*p* = 0.030).

**Conclusions:**

Depressive symptoms measured with the total BDI scores associated with sleep impairment in both perimenopausal and postmenopausal women. In younger women, specific BDI factors revealed minor associations, suggesting that the type of sleep impairment can vary in relation to different depressive features. Our data indicate that associations between sleep and depressed mood may change in conjunction with hormonal milestones.

## Background

Depressive symptoms are more common in women than in men [1,2]. Mood disorders, and in particular major depressive disorder (MDD), are also known to be twice as common in women as in men [3]. In addition, the onset, course and symptoms of depression differ between genders, as women are more likely to suffer from atypical depression, psychomotor retardation, anxiety/somatic symptoms, psychological distress, increased appetite and weight gain than men [2-6].

Although this gender difference persists throughout the female life-span, it seems to vary according to the reproductive state. The risk of depressive symptoms and that of disorders are higher during certain reproductive events such as the premenstrual phase, pregnancy and postpartum [7], and the transition to menopause [8,9]. In particular, during the climacterium there is an increased risk of experiencing low mood, irritability and difficulties in concentration, even after controlling for possible confounding factors [10-12], and up to 70–90% of women report depressive symptoms [13].

Depressive symptoms and disorders are often accompanied by sleep disturbances. Indeed, sleep is typically disrupted in depressed subjects, with an abnormal amount and/or an abnormal distribution of rapid-eye-movement (REM) sleep and non-REM sleep [14], older individuals being more likely to suffer from middle or late insomnia and younger ones from hypersomnia [15-17]. Sleep impairment has been claimed as a prodromal symptom of depression, and some sleep abnormalities are considered as specific markers for MDD, such as impairment in sleep continuity, a lack of inhibition of REM sleep, and reduction in slow-wave sleep (SWS) [14,18]. Reciprocally, subjects suffering from insomnia or other sleep disturbances seem to be at a higher risk of developing MDD [19] and having recurrent depressive episodes [20]. However, whether sleep disturbances are part of the pathophysiology of depression or its consequences, it is not well understood [21,22]. Possibly, sleep disturbances and other psychiatric disorders share a common multifactorial etiology here [23].

Age and fluctuations of female sex hormones are likely to be part of this multifactorial etiology. Age is associated with a reduction of REM sleep and SWS [24-26], with an increase in time spent in lighter sleep stages and a general decrease in sleep efficiency especially in women [27]. On the other hand, aging is tightly inter-related with the transition to menopause, which in turn is characterized by an intense decrease of sex hormone levels. Women do commonly report sleep difficulties with an impaired subjective sleep quality and, to a lesser extent, also an impaired objective sleep quality during perimenopause and in postmenopause [28-32]. Recently, Orff and colleagues [33] found that age and the reproductive status affect a different set of objective sleep parameters in women. One important study to focus on the relationship between reproductive state, sex hormone levels, sleep and depression [34], came to the conclusion that the association between hormonal changes and menopause could contribute to the increase in the sleep-endocrine alterations that are typically found in depression.

Given the complexity of the inter-relationship between mood, sleep, age and reproductive state, and the lack of clear evidence on this issue, the aim of our study was to evaluate the relationship between mood and sleep among healthy women in different reproductive states. We hypothesized that the relationship between lower mood and lower sleep quality would strengthen during and after the menopause, thereby partly explaining the increase in mood and sleep symptoms typically found in these periods. Mood was assessed not only with a conventional total depression score, but also with a factorial design for a detailed evaluation of the relationships in order to identify specific subcategories, if any, of depressive symptoms.

## Methods

### Subjects and study design

Altogether 61 women took part in the study: 11 younger women (age range 20–26 years), 21 perimenopausal women (age range 43–51 years) and 29 postmenopausal women (age range 58–71 years). This study was a part of a larger study investigating the effects of menopause on sleep and cognition [35]. In order to avoid the possible confounding effects of health-illness-related factors, healthy women only were selected for the study. Younger women were recruited via announcements at the University of Helsinki and studied in the Sleep Research Unit of the Institute of Biomedicine, University of Helsinki, Finland. The perimenopausal and postmenopausal women were enrolled through announcements in the local newspapers in the area of Turku and the studies were conducted in the Sleep Research Unit of the University in Turku, Finland. The Turku Sleep Research Unit served as the supervising and monitoring unit of the study. Perimenopausal status was defined by the serum follicle stimulating hormone (FSH) level (<23 IU/mL) and an ongoing regular or irregular menstrual cycle. Postmenopause was determined by age (≥58 years) and at least 12 months of amenorrhea. None of the perimenopausal and postmenopausal women had used any hormone therapy (HT) in at least 12 months prior to or during the study, with the exception of one perimenopausal woman, in whom the washout period was five months. The younger women were all currently taking oral contraceptives (OCs) (ethinyl estradiol 20 ug + desogestrel 0.15 mg, Mercilon^©^, Organon, Oss, the Netherlands). Perimenopausal and younger women were examined during the first days of their menstrual cycle at the follicular phase.

Presence of a mental, cardiovascular (except drug-treated balanced hypertension), endocrine (except drug-treated balanced hyperlipidemia), pulmonary, neurological or specific sleep disorder or malignancies was one of the exclusion criteria. Also women suffering from other conditions possibly affecting sleep (e.g. fibromyalgia or anemia) were excluded. Other exclusion criteria included the use of medication with central nervous system effects, alcohol abuse, smoking and excessive caffeine intake (>5 cups of coffee per day). All women had regular sleep-wake schedules and normal levels of blood hemoglobin, leucocytes, thrombocytes and serum thyrotropin. All women gave written informed consent after oral and written information. The study was approved by the Ethics Committee of Turku University Hospital and the University of Helsinki.

### Mood and sleep assessments

The presence of current depressive symptoms was assessed via the 21-item Beck Depression Inventory (BDI) [36]. As the study sample consisted of healthy women of different age ranges, in addition to considering the BDI total score, a factor analysis was performed to identify the factors that could best explain the BDI items (Cronbach’s alpha = 0.75) in the whole sample. Because depressive disorders are notoriously multidimensional and characterized by different symptomatic patterns according to a number of correlates (including age and gender), a factor analysis was chosen as an approach to address depressive subcomponents. Four factors were identified and, on the basis of their most weighted loadings, named as “punishment/guilty feelings” (F1: guilty feelings, punishment feelings and self-dislike/disappointment), “dissatisfaction” (F2: loss of pleasure, worries about one’s health, feeling of looking ugly, sadness, lost interest in people), “depressive-emotional” (F3: lost appetite, crying, pessimism, sadness and feeling of worthlessness), and “depressive-somatic” (F4: indecisiveness, changed sleep pattern, lost interest in sex, past failure, impaired working capability, tiredness and irritability) patterns (Table 1). Items with no (#9: suicidal ideation) or only one (#19: weight loss) positive answer were excluded from the analyses.

**Table 1:** BDI factor analysis^*^

	**F1**	**F2**	**F3**	**F4**	**Sumsqr**
**#1 sadness**	0.099	**0.445**	**0.304**	0.268	0.372
**#2 pessimism**	−0.027	0.032	**0.306**	0.213	0.141
**#3 past failure**	0.243	0.224	0.039	**0.493**	0.354
**#4 loss of pleasure**	−0.178	**0.980**	0.025	−0.005	0.993
**#5 guilty feelings**	**0.390**	0.175	0.258	0.227	0.301
**#6 punishment feelings**	**0.928**	0.286	−0.005	−0.239	1.000
**#7 self-dislike**	**0.295**	−0.073	−0.034	0.199	0.133
**#8 worthlessness**	−0.022	0.172	**0.289**	0.147	0.135
**#10 crying**	0.062	0.051	**0.322**	−0.031	0.111
**#11 irritability**	0.012	0.207	0.099	**0.313**	0.151
**#12 lost interest in people**	0.168	**0.375**	0.183	0.060	0.206
**#13 indecisiveness**	−0.033	0.133	0.158	**0.582**	0.383
**#14 looking ugly**	0.115	**0.445**	−0.019	0.238	0.269
**#15 impaired work capability**	0.074	−0.149	−0.036	**0.482**	0.262
**#16 changed sleep pattern**	0.153	0.087	0.218	**0.554**	0.385
**#17 fatigue or tiredness**	0.003	0.175	−0.105	**0.476**	0.269
**#18 lost appetite**	−0.033	−0.110	**0.984**	−0.104	0.993
**#20 worries about health**	0.295	**0.486**	0.006	0.099	0.333
**#21 lost interest in sex**	−0.077	0.377	0.058	**0.521**	0.423
**Sumsqr**	1.372	2.211	1.544	2.088	

*Items with no (# 9: suicidal ideation) or one (# 19: weight loss) positive answer excluded from the analysis. Weighted loadings at each factor in boldface.

*Abbreviations*: *F1* punishment/guilty feelings, *F2* dissatisfaction, *F3* depressive-emotional symptoms, *F4* depressive-somatic symptoms.

Subjective insomnia (a sum score, with the range of 5–25) during the past three months was evaluated using the Basic Nordic Sleep Questionnaire (BNSQ) [37], with lower scores referring to lower levels of sleeping problems. The subjects kept a sleep diary during the three weeks before and one week after the study to verify their sleep-wake schedules: all women had regular sleep-wake schedules (10:00 – 11:00 pm to 6:00 – 7:00 am). The women spent one adaptation night (7:30 pm – 8:00 am, time in bed 11:00 pm – 7:00 am) in the sleep laboratory. In the following morning, a venous blood sample was taken for baseline serum FSH and estradiol (E2) measurements. In the following evening, the women returned to the laboratory at 7:30 pm for the baseline sleep recording. The all-night polysomnography (PSG) recordings consisted of continuous monitoring via electroencephalograms (EEG), electro-oculograms (EOG), a mandibular electromyogram (EMG) and an electrocardiogram (ECG, Embla®, Medcare Flaga hf. Medical devices, Reykjavik, Iceland). Two EEG-channels (C3/A2, C4/A1) were used in younger women, while four EEG-channels (C3/A2, C4/A1, O1/A2 and O2/A2) were used in perimenopausal and postmenopausal women. Sleep stages were visually scored in 30-s epochs according to conventional criteria [38] by the same scorer (NK) and controlled by a senior scorer (PP-K). For the purpose of this study, three groups of sleep variables were considered as follows.

1. general sleep: sleep latency (period from lights off to sleep onset, defined as the appearance of three consecutive epochs of S1 or the first epoch of any other stage), total sleep time [sum of time spent in Stage 1 (S1), Stage 2 (S2), SWS, REM sleep and movement time (MT)], sleep efficiency (the percentage of total sleep time out of time in bed) and number of arousals (EEG α-activity for at least three seconds [39]);

2. REM sleep: REM latency (the time from sleep onset to the first 30 s of the REM stage), REM percentage (the percentage of total time in bed, from lights off to lights on) and REM awakenings (the number of times entering wake stage from REM sleep);

3. SWS: SWS latency (the time from sleep onset to the first 30 s of the SWS), SWS percentage (the percentage of total time in bed, from lights off to lights on), SWS awakenings (the number of times entering wake stage from SWS) and total slow wave activity in NREM-sleep (SWA, 0.75–4 Hz) [40].

### Statistical analysis

One perimenopausal and one postmenopausal woman were excluded from the analyses because of missing data. The normality of the distributions was tested with the Shapiro-Wilk test. Since the distributions of some variables were skewed, non-parametric Kruskal-Wallis test was performed to compare means between the three groups, using reproductive state as grouping variable (younger, perimenopausal or postmenopausal). When a significant difference was found, the pair-wise differences between each group were tested with Student’s *t*-test (in the case of normal distribution) or Mann–Whitney test, as appropriate. The results were corrected according to the Bonferroni procedure. A *p*-value of < 0.05 was considered significant.

A factor analysis was performed to identify the factors that best explained the variability within the BDI items. The factor matrix was computed according to the maximum likelihood principle and employing the algorithm presented by Jöreskog [41]. The standard orthogonal Varimax rotation was used to examine the degree of correlation among factors. The reliability estimates were computed according to the principles given by Cronbach [42] and the general measurement framework introduced by Tarkkonen [43] and developed further by Vehkalahti [44] using the RELIAB module of the Survo MM program [45].

Multivariable analysis was performed using generalized linear models to test the associations between sleep variables *vs.* BDI total score and each of the identified BDI factors. The analyses were performed separately on each group. The results were further controlled for age. Data were visually checked for outliers, defined as cases falling outside more than 1.5 times the interquartile range. All the multivariable analyses were repeated after exclusion of the identified outliers. Additionally, given the possible bias brought by the well-known tight inter-relationship between sleep and depressive symptoms, multivariable analyses were repeated after exclusion of the sleep-related items (#16 and #17) from computation of the BDI total score. Descriptive and multivariable analyses were performed using SPSS/PASW software (version 18.0) (SPSS Inc., Chicago, IL, USA).

## Results

Background characteristics of the participants are reported in Table 2. All the younger women had a BDI total score lower than 10 (range 0–5), indicating no depressive symptoms; one perimenopausal woman (BDI = 11) and 6 postmenopausal women had a BDI total score ranging between 10 and 18 (BDI = 10 in two, BDI = 11 in one, BDI = 13 in two and BDI = 15 in one), indicating mild to moderate depressive symptoms. Postmenopausal women had higher mean BDI total score than younger women (6.3, SD 3.9 *vs.* 1.8, SD 1.7; *p* = 0.003), while no difference emerged between perimenopausal and younger women (4.1, SD 3.3 *vs.* 1.8, SD 1.7; *p* = 0.147), or between perimenopausal and postmenopausal women (4.1, SD 3.3 *vs.* 6.3, SD 3.9; *p* = 0.147). The three groups differed in sleep as follows: when comparing with either perimenopausal or postmenopausal women, the younger women had longer total sleep time, better sleep efficiency, a greater SWS percentage, higher total SWA, less REM awakenings and less SWS awakenings. They also had longer REM latency than postmenopausal women. Additionally, younger women had lower insomnia scores at the BNSQ than both perimenopausal and postmenopausal women. Compared to perimenopausal women, postmenopausal women had higher BNSQ insomnia score. Results of pair-wise comparisons of the sleep variables in younger, perimenopausal and postmenopausal women have been published earlier in detail [40]; see also Additional file 1.

**Table 2:** Background characteristics of the participants

	**Younger (n =11)**	**Perimenopausal (n = 20)**	**Postmenopausal (n = 28)**
	**mean (SD)**		
**Age** (years)	23.1 (1.6)	47.7 (2.3)	63.3 (3.6)
**FSH** (U/L)	3.8 (2.8)	10.6 (4.8)	76.2 (29.2)
**E2** (pml/L)	91.8 (87.3)	327.2 (330.2)	31.2 (10.6)
**BMI** kg/m^2^	23.1 (3.1)	23.9 (2.4)	27.2 (4.2)
**BDI** score (total)	1.8 (1.7)	4.1 (3.3)	6.3 (3.9)
**F1** punishment/guilty feelings	−0.26 (0.08)	0.18 (1.26)	0.01 (1.0)
**F2** dissatisfaction	−0.48 (0.06)	0.04 (1.08)	0.10 (1.05)
**F3** depressive-emotional symptoms	0.20 (1.64)	−0.20 (0.14)	0.07 (1.07)
**F4** depressive-somatic symptoms	−0.62 (0.38)	−0.21 (0.70)	0.38 (1.02)

Note: BDI = Beck depression inventory; BMI = body mass index; E2 = estradiol; FSH = follicle stimulating hormone.

### BDI factors and insomnia

Higher BDI total score was associated with higher BNSQ insomnia score in perimenopausal women (B = 0.457; 95% CI = 0.139 to 0.775; *p* = 0.005), also after controlling for age. However, the significance was lost when the sleep-related items (#16 and #17) were removed from the BDI total score (B = 0.256; 95% CI = −0.017 to 0.530; *p* = 0.066). Higher scores at the depressive-emotional pattern (*p* = 0.043 and *p* = 0.011) in younger and perimenopausal women, at the depressive-somatic pattern (*p* = 0.036) in perimenopausal women, and at the punishment/guilty feelings factor (*p* = 0.010) in postmenopausal women were associated with higher BNSQ insomnia score. All the associations were maintained after removing of potential outliers. Further associations emerged after age adjustment: higher dissatisfaction score (*p* = 0.023 and *p* = 0.042) was associated with higher insomnia score in younger and perimenopausal women, while the association with depressive-somatic symptoms lost the significance (Table 3).

**Table 3:** **Associations between BDI factors *vs. *** **subjective sleep quality (BNSQ insomnia)**

	**Punishment/guilty feelings (F1)**				**Dissatisfaction (F2)**			**Depressive-emotional (F3)**			**Depressive-somatic (F4)**		
	**B**		**95% CI**	***p***	**B**	**95% CI**	***p***	**B**	**95% CI**	***p***	**B**	**95% CI**	***p***
**Younger women**													
**BNSQ insomnia**	**simple**	−0.005	−0.025 to 0.015	0.651	−0.011	−0.023 to 0.001	0.081	*0.366*	*0.011 to 0.720*	*0.043*	−0.039	−0.133 to 0.055	0.416
	**age-adjusted**	−0.009	−0.028 to 0.011	0.385	*−0.014*	*−0.025 to −0.002*	*0.023*	*0.442*	*0.103 to 0.781*	*0.011*	−0.059	−0.149 to 0.030	0.194
**Perimenopausal women**													
**BNSQ insomnia**	**simple**	−0.062	−0.205 to 0.081	0.394	0.105	−0.011 to 0.221	0.077	*0.018*	*0.004 to 0.032*	*0.011*	*0.078*	*0.005 to 0.151*	*0.036*
	**age-adjusted**	0.046	−0.134 to 0.226	0.618	*0.159*	*0.006 to 0.312*	*0.042*	*0.025*	*0.007 to 0.043*	*0.006*	0.052	−0.045 to 0.149	0.296
**Postmenopausal women**													
**BNSQ insomnia**	**simple**	*0.123*	*0.029 to 0.216*	*0.010*	−0.054	−0.161 to 0.053	0.323	0.003	−0.109 to 0.115	0.955	0.088	−0.013 to 0.189	0.086
	**age-adjusted**	*0.126*	*0.035 to 0.217*	*0.007*	−0.056	−0.163 to 0.051	0.306	0.006	−0.104 to 0.117	0.913	0.086	−0.014 to 0.187	0.091

Associations between each BDI factor and BNSQ insomnia score were tested in each reproductive group separately via generalized linear models. Potential outliers included in the models.

Significant items are in italic.

Note: BDI = Beck depression inventory; BNSQ = basic Nordic sleep questionnaire; 95% CI = 95% confidence intervals.

### BDI factors and general sleep

In younger women higher BDI total score was associated with lower amount of arousals (B = −0.042; 95% CI = −0.079 to −0.005; *p* = 0.026), and in perimenopausal women with lower sleep efficiency (B = −0.159; 95% CI = −0.296 to −0.023; *p* = 0.022) and shorter total sleep time (B = −0.033; 95% CI = −0.061 to −0.004; *p* = 0.024), also after controlling for age. The associations with sleep efficiency (B = −0.097; 95% CI = −0.210 to 0.016; *p* = 0.002) and total sleep time (B = −0.020; 95% CI = −0.043 to 0.004; *p* = 0.100) in perimenopausal women were lost after removing the sleep-related items (#16 and # 17) from the BDI total score. In perimenopausal women higher score at the depressive-somatic pattern was associated with longer sleep latency (*p* = 0.032); however, the association was lost after controlling for age or after removing the outliers. No associations were found in postmenopausal women (Table 4). However, when outliers were removed, significant associations emerged between higher punishment/guilty feeling scores and shorter sleep time (B = −0.012; 95% CI = −0.021 to −0.003; *p* = 0.008) and lower sleep efficiency (B = −0.058; 95% CI = −0.100 to −0.015; *p* = 0.008) in postmenopausal women.

**Table 4:** **Associations**^**^ **^**between BDI factors *vs. *** **general sleep variables**

	**Punishment/guilty feelings (F1)**			**Dissatisfaction (F2)**			**Depressive-emotional symptoms (F3)**			**Depressive-somatic symptoms (F4)**		
	**B**	**95% CI**	***p***	**B**	**95% CI**	***p***	**B**	**95% CI**	***p***	**B**	**95% CI**	***p***
**Younger women**												
**Sleep latency**	0.002	−0.003 to 0.007	0.374	0.002	−0.002 to 0.005	0.384	−0.035	−0.139 to 0.068	0.504	0.011	−0.012 to 0.035	0.346
**Sleep efficiency**	0.002	−0.007 to 0.010	0.672	0.001	−0.004 to 0.007	0.666	−0.039	−0.210 to 0.132	0.655	0.009	−0.030 to 0.049	0.644
**Total sleep time**	0.000	−0.001 to 0.002	0.687	0.000	−0.001 to 0.001	0.666	−0.008	−0.043 to 0.027	0.649	0.002	−0.006 to 0.010	0.655
**Number of arousals**	0.000	−0.002 to 0.002	0.695	0.000	−0.001 to 0.002	0.675	−0.015	−0.056 to 0.027	0.487	−0.001	−0.011 to 0.009	0.863
**Perimenopausal women**												
**Sleep latency**	−0.009	−0.044 to 0.026	0.611	−0.010	−0.039 to 0.020	0.521	0.001	−0.003 to 0.004	0.793	*0.019*	*0.002 to 0.037*	*0.032*
**Sleep efficiency**	−0.004	−0.063 to 0.055	0.896	−0.028	−0.077 to 0.022	0.276	−0.005	−0.011 to 0.001	0.075	−0.023	−0.054 to 0.009	0.159
**Total sleep time**	−0.001	−0.013 to 0.012	0.905	−0.006	−0.016 to 0.005	0.295	−0.001	−0.002 to 0.000	0.082	−0.005	−0.011 to 0.002	0.155
**Number of arousals**	−0.002	−0.014 to 0.011	0.806	−0.005	−0.016 to 0.005	0.290	0.000	−0.001 to 0.001	0.823	0.005	−0.002 to 0.011	0.167
**Postmenopausal women**												
**Sleep latency**	0.019	−0.006 to 0.043	0.135	−0.014	−0.041 to 0.012	0.281	0.016	−0.011 to 0.043	0.245	0.002	−0.024 to 0.028	0.865
**Sleep efficiency**	−0.028	−0.059 to 0.003	0.079	0.014	−0.020 to 0.048	0.405	−0.020	−0.054 to 0.014	0.254	0.003	−0.030 to 0.037	0.843
**Total sleep time**	−0.006	−0.012 to 0.001	0.080	0.003	−0.004 to 0.010	0.409	−0.004	−0.011 to 0.003	0.252	0.001	−0.006 to 0.008	0.845
**Number of arousals**	−0.001	−0.006 to 0.004	0.767	0.002	−0.003 to 0.008	0.399	−0.002	−0.008 to 0.003	0.432	−0.005	−0.010 to 0.000	0.068

Associations between each BDI factor and general sleep variables were tested in each reproductive group separately via generalized linear models. Potential outliers included in the models.

Significant items are in italic.

^**^**^not age-adjusted.

Note: BDI = Beck depression inventory; 95% CI = 95% confidence intervals.

### BDI factors and REM sleep

Higher BDI total score associated with a greater REM percentage in postmenopausal women (B = 0.301; 95% CI = 0.049 to 0.553; *p* = 0.019) also after controlling for age, while no associations were found in the other groups. However, the association was lost when the sleep-related items (#16 and #17) were removed from the BDI total score (B = 0.214; 95% CI = −0.032 to 0.459; *p* = 0.088). Lower scores at the punishment/guilty feelings (*p* = 0.005), dissatisfaction (*p* < 0.001) and depressive-somatic pattern (*p* = 0.001) and higher score at the depressive-emotional pattern (*p* = 0.002) were associated with higher amount of REM awakenings in younger women. Higher dissatisfaction (*p* = 0.017) score related to longer REM latency in perimenopausal women. Higher scores at the depressive-somatic pattern (*p* = 0.005) related to a greater REM percentage in postmenopausal women (Table 5), but the association was lost after removing of the outliers. All the above-mentioned associations, with the only exception of the one between lower score at the depressive-somatic pattern and higher amount of REM awakenings in younger women, were maintained after controlling for age.

**Table 5:** **Associations**^**^ **^**between BDI factors *vs. *** **REM sleep variables**

	**Punishment/guilty feelings (F1)**			**Dissatisfaction (F2)**			**Depressive-emotional symptoms (F3)**			**Depressive-somatic symptoms (F4)**		
	**B**	**95% CI**	***p***	**B**	**95% CI**	***p***	**B**	**95% CI**	***p***	**B**	**95% CI**	***p***
**Younger women**												
**REM latency**	0.001	0.000 to 0.002	0.145	0.001	0.000 to 0.001	0.178	−0.013	−0.039 to 0.012	0.316	0.004	−0.001 to 0.010	0.127
**REM %**	−0.002	−0.014 to 0.009	0.689	0.000	−0.007 to 0.008	0.917	−0.045	−0.276 to 0.187	0.704	−0.007	−0.061 to 0.047	0.790
**REM awakenings**	*−0.047*	*−0.080 to −0.014*	*0.005*	*−0.037*	*−0.056 to −0.017*	*<0.001*	*1.005*	*0.357 to 1.652*	*0.002*	*−0.247*	*−0.391 to −0.103*	*0.001*
**Perimenopausal women**												
**REM latency**	0.002	−0.015 to 0.020	0.787	*0.016*	*0.003 to 0.030*	*0.017*	0.001	0.000 to 0.003	0.148	−0.005	−0.015 to 0.004	0.290
**REM %**	0.036	−0.053 to 0.124	0.428	−0.026	−0.102 to 0.051	0.509	−0.004	−0.013 to 0.006	0.451	−0.019	−0.068 to 0.031	0.459
**REM awakenings**	−0.188	−0.406 to 0.030	0.091	0.005	−0.195 to 0.204	0.961	−0.007	−0.032 to 0.018	0.568	−0.011	−0.140 to 0.118	0.865
**Postmenopausal women**												
**REM latency**	−0.001	−0.016 to 0.013	0.855	−0.008	−0.023 to 0.006	0.269	−0.001	−0.017 to 0.015	0.895	0.003	−0.012 to 0.018	0.713
**REM %**	0.004	−0.066 to 0.074	0.911	0.022	−0.052 to 0.095	0.558	0.038	−0.036 to 0.112	0.317	*0.091*	*0.028 to 0.154*	*0.005*
**REM awakenings**	0.025	−0.092 to 0.143	0.672	0.064	−0.057 to 0.186	0.297	−0.052	−0.177 to 0.073	0.414	−0.076	−0.193 to 0.040	0.200

Associations between each BDI factor and REM sleep variables were tested in each reproductive group separately via generalized linear models. Potential outliers included in the models.

Significant items are in italic.

^**^**^not age-adjusted.

Note: BDI = Beck depression inventory; 95% CI = 95% confidence intervals; REM = rapid eye movement.

### BDI factors and SWS

No associations were found between BDI total score and any of the SWS variables. However, in younger women higher scores at the dissatisfaction (*p* = 0.001) and depressive-somatic pattern (*p* = 0.025), and lower score at the depressive-emotional pattern (*p* = 0.001) associated with a greater SWS percentage. Moreover, lower scores at the punishment/guilty feelings (*p* = 0.036), dissatisfaction (*p* = 0.001) and depressive-somatic pattern (*p* = 0.009), and higher score at the depressive-emotional pattern (*p* = 0.003) associated with higher amount of SWS awakenings. Higher score at the depressive-somatic pattern (*p* = 0.030) score related to longer SWS latency in postmenopausal women (Table 6). All the associations, except those of the depressive-somatic pattern in younger women, maintained their significance after controlling for age. When removing the outliers, a higher score at punishment/guilty feelings, but not at the depressive-somatic pattern, was associated with longer SWS latency (B = 0.095; 95% CI = 0.035 to 0.155; *p* = 0.002) in postmenopausal women.

**Table 6:** **Associations**^**^ **^**between BDI factors *vs. *** **SWS variables**

	**Punishment/guilty feelings (F1)**			**Dissatisfaction (F2)**			**Depressive-emotional symptoms (F3)**			**Depressive-somatic symptoms (F4)**		
	**B**	**95% CI**	***p***	**B**	**95% CI**	***p***	**B**	**95% CI**	***p***	**B**	**95% CI**	***p***
**Younger women**												
**SWS latency**	0.000	−0.008 to 0.008	0.936	−0.001	−0.006 to 0.005	0.827	0.024	−0.142 to 0.189	0.778	0.000	−0.039 to 0.039	0.997
**SWS %**	0.011	−0.002 to 0.023	0.093	*0.012*	*0.005 to 0.018*	*0.001*	*−0.344*	*−0.542 to −0.147*	*0.001*	*0.062*	*0.008 to 0.117*	*0.025*
**SWS awakenings**	*−0.106*	*−0.205 to −0.007*	*0.036*	*−0.096*	*−0.152 to −0.040*	*0.001*	*2.701*	*0.913 to 4.490*	*0.003*	*−0.585*	*−1.024 to −0.146*	*0.009*
**SWA total**	0.000	−0.001 to 0.000	0.455	0.000	0.000	0.697	0.000	0.000	0.477	−0.001	−0.003 to 0.001	0.464
**Perimenopausal women**												
**SWS latency**	−0.009	−0.041 to 0.023	0.580	−0.017	−0.044 to 0.009	0.196	−0.002	−0.005 to 0.001	0.262	0.003	−0.014 to 0.021	0.697
**SWS %**	−0.022	−0.117 to 0.073	0.656	0.028	−0.052 to 0.109	0.492	0.005	−0.005 to 0.015	0.338	0.020	−0.032 to 0.072	0.442
**SWS awakenings**	−0.069	−0.437 to 0.299	0.714	0.052	−0.264 to 0.367	0.749	0.015	−0.025 to 0.054	0.467	0.107	−0.092 to 0.306	0.292
**SWA total**	−0.002	−0.008 to 0.003	0.441	−0.002	−0.007 to 0.003	0.396	0.000	−0.001 to 0.000	0.188	−0.001	−0.004 to 0.002	0.542
**Postmenopausal women**												
**SWS latency**	0.015	−0.007 to 0.037	0.181	−0.009	−0.033 to 0.014	0.427	0.001	−0.023 to 0.025	0.940	*0.024*	*0.002 to 0.045*	*0.030*
**SWS %**	−0.026	−0.075 to 0.024	0.305	0.006	−0.047 to 0.058	0.835	−0.030	−0.083 to 0.023	0.271	−0.040	−0.089 to 0.010	0.115
**SWS awakenings**	−0.147	−0.359 to 0.065	0.174	0.044	−0.185 to 0.273	0.708	−0.025	−0.210 to 0.260	0.833	−0.168	−0.383 to 0.046	0.124
**SWA total**	−0.009	−0.021 to 0.002	0.122	0.002	−0.010 to 0.015	0.703	−0.009	−0.021 to 0.004	0.185	−0.009	−0.021 to 0.003	0.133

Associations between each BDI factor and SWS variables were tested in each reproductive group separately via generalized linear models. Potential outliers included in the models.

Significant items are in italic.

^**^**^not age-adjusted.

Note: BDI = Beck depression inventory; 95% CI = 95% confidence intervals; SWA = slow wave activity SWS = slow wave sleep.

(The associations between mood symptoms and sleep quality in the three groups are illustrated in Additional files 2: Figure S1, Additional file 3: Figure S2 and Additional file 4: Figure S3).

## Discussion

Associations between sleep stages or sleep quality and the clinically subthreshold depressive symptoms were evident both in perimenopausal and postmenopausal women. Higher depression scores associated with a lower subjective sleep quality (a higher insomnia score) as well as a lower objective sleep quality (a lower sleep efficiency and a shorter total sleep time), especially in perimenopausal women. Also, postmenopausal women with a higher BDI score had more REM sleep. On the contrary, the total BDI score was not sensitive enough to detect any significant associations with sleep in younger women.

To identify specific subgroups of depressive symptoms, factor analysis yielded a dual pattern of depression-with-sleep associations in younger women with respect to the SWS amount and SWS or REM awakenings: more depressive-emotional symptoms were associated with a smaller SWS percentage and with more SWS awakenings and more REM sleep awakenings, whereas, vice versa, the associations towards the opposite directions were found concerning punishment, dissatisfaction and depressive-somatic symptoms. In perimenopausal women more dissatisfaction was associated with a longer REM latency, and more depressive-somatic symptoms with a worse sleep quality (higher BNSQ score and longer sleep latency). In addition, more depressive-somatic symptoms were associated with a greater REM percentage and a longer SWS latency in postmenopausal women. When the outliers or the sleep-related items (#16 and #17) were removed from the analyses, most of the associations with depressive somatic symptoms in perimenopausal and postmenopausal women were lost; rather, more punishment/guilty feelings were found to be associated with a worse sleep quality (higher scores of the subjective insomnia on BNSQ, a lower sleep efficiency, a shorter total sleep time, and a longer SWS latency) in postmenopausal women.

Previous studies focusing on the relationship between mood and sleep have typically used either diagnostic categories or sum scores of different depression assessment tools [46-49]. In the current study, we used the BDI total score which, however, produced limited associations, probably because of the generally good mental health status of our participants. The total BDI score was a sensitive enough index for detecting some previously reported associations between mood and sleep [33], especially in perimenopausal women, and to a lesser extent, in postmenopausal women. In specific, a relationship with the subjective sleep quality, known to be impaired in the context of depressive symptoms [33], was evident only in perimenopausal women. Additionally, a relationship between depressive symptoms and impairment in the objective sleep quality emerged both in perimenopausal (a shorter sleep time and a decreased sleep efficiency) and postmenopausal (a greater REM percentage) women, both findings being specific markers for depression [18,33]. However, it is of note that these associations lost their significance when the sleep-related items (#16 and #17) were removed from the analyses, further highlighting the tight inter-relationship between objective sleep impairment and depressive symptoms, and thus limiting any causal inference. The fact that the significance was lost after removing the sleep-related items may also suggest that the above-mentioned findings were mainly expression of poor sleep rather than of a true association between poor sleep and depressive symptoms in these reproductive groups.

The literature is rather consistent in reporting associations between depressive symptoms and sleep impairment already in young age [50,51]. However, in our study only one connection between the total BDI score and sleep was found in younger women, where hypersomnia rather than sleep impairment was connected with depressive symptoms. This could be due to the overall normal mood and sleep quality in young women. Because of these limited findings, we further performed a factor analysis, which grouped related BDI items into four specific categories with different depressive dimensions. As said, this was done in an attempt to better identify the different sub-components that are likely to characterize paucisymptomatic depressed women at different age and reproductive stages. When analyzing the associations between the BDI factors and sleep, a dual pattern of associations was consistently displayed in younger women: either the one between the depressive-emotional factor and more disrupted sleep, or that between punishment, dissatisfaction and depressive-somatic factors and less disrupted sleep. The depressive-emotional factor, which included feelings of sadness, hopelessness and worthlessness, was associated with a smaller SWS percentage and an increased number of awakenings from both SWS and REM sleep. The other factors, i.e. punishment (including also self-dislike), dissatisfaction (including loss of pleasure and worries about health), and depressive-somatic (expression of functional impairment) symptoms, had these associations in the opposite direction. Thus, it seemed that the depressive-emotional factor revealed a finding typical of depression, where depressed persons report (and have) a reduced sleep quality or insomnia [14,21]. On the other hand, the other BDI factors were associated with more hypersomnic features, suggesting that different depressive symptoms may be related to different dimensions of sleep impairment. In younger women more REM awakenings were associated with more depressive emotional symptoms, but with lower scores on the other depressive factors. This apparent contradiction could be explained by the fact that a high amount of REM sleep and sleep fragmentation are specific markers for depression. Emotional symptoms are likely to be the main dimension of depression in younger women, while other symptoms, including the somatic ones, are a major depressive feature in older individuals [52,53]. In this context, it is possible that the increased number of awakenings from REM sleep, expression of more fragmented sleep, results associated with the most significant depressive factor in young women (the emotional dimension) rather than with the remaining dimensions. This would be in line with the cognition of a tight inter-relation between sleep (in particular REM sleep) and the emotional processing [54]. Another possible explanation to the apparent better sleep quality in relation to punishment, dissatisfaction and depressive-somatic factors in younger women might be that they have retained the ability to cope with depressive symptoms through improving their sleep quality. This hypothesis is partly supported by the findings by Talamini et al. [55]. In a sample of young healthy individuals (mean age 20 years, 72% women) the authors found a bimodal pattern of response to an externally induced distress in terms of sleep quality. They concluded that, due to individual differences, some subjects may respond adaptively, i.e. improving their quality of sleep, while other individuals may respond in a maladaptive manner, i.e. with a reduction of their sleep quality. Based on their results, the authors suggested that a high SWS percentage may be associated with higher levels of “emotional dissipation” during sleep, while an increased number of awakenings to poor emotional dissipation. This is partly in line with our findings, where more (REM and SWS) awakenings and a smaller SWS percentage were related to higher emotional depressive symptoms, but lower scores on other depressive dimensions.

As sleep and its quality are known to deteriorate with age (including aging of the hypothalamus-pituitary-ovarian system, i.e. with the transition into menopause), it can be hypothesized that this ability also deteriorates with, or is altered by aging. In fact, this dual pattern in the depression-sleep associations was not found among perimenopausal or postmenopausal women. It is therefore possible that these different patterns of associations reflect different symptomatic dimensions of depression at different ages. Based on our results, it seems that the emotional dimension is especially relevant in younger women, while the somatic component is transiently more evident in the perimenopausal group, and the affective dimension more specifically related to punishment and guilty feelings becomes relevant in postmenopausal women. However, as mentioned above, in this study the younger women in particular had low BDI scores, suggesting very limited and mild depressive symptoms. However, even subthreshold depression is known to be associated with a poor quality of life [56], and adolescents with subthreshold depressive conditions have not only an impaired functioning and higher levels of suicidality [57], but also a high risk of developing a major depressive episode [58].

Only in perimenopausal women, a connection between more dissatisfaction and a longer REM latency was found. This is partly in line with previous findings of a prolonged sleep latency in depressed subjects [14]. During the perimenopausal period women are prone to experience a variety of depressive and somatic symptoms [59-61] that may equally belong to the four different BDI factors identified in our study. Therefore, the total BDI score was more sensitive in identifying the associations with impaired sleep quality. In addition, a mismatch between subjective and objective sleep impairment in association with depressive symptoms and disorders has been reported during the menopausal transition, when the common report of sleep complaints and dissatisfaction with sleep not necessarily correlates with objective sleep impairment [62]. Furthermore, factors other than depression may have a more significant role in causing the sleep impairment typical for the perimenopausal period. For instance, gradually decreasing female sex hormone levels may directly affect sleep [63], and climacteric vasomotor symptoms are known to be associated with sleep impairment [30,32,64-67], independently of mood symptoms. In fact, not only depressive symptoms but also depressive disorders in perimenopausal and postmenopausal women could be due to the vasomotor climacteric symptoms, which impair sleep quality and quality of life in general, resulting in compromised psychological well-being and depression [68]. However, it is of note that some studies have found an antidepressant effect of hormone therapy in menopausal women irrespective of their vasomotor symptoms [69].

In our sample of postmenopausal women higher depressive-somatic symptoms were associated with more REM sleep and a longer SWS latency. Somatic complaints, which may in turn induce depression and impaired sleep, become more frequent with age, especially in women. Women complain more often than men about somatic symptoms associated with psychological distress [70], and the rates of “somatic depression”, albeit low, are higher in women than in men [70,71]. As previously mentioned for the perimenopausal women, vasomotor symptoms tend to be associated with both depression and sleep disturbances [30,32,72] and may account for this finding. These associations with the depressive-somatic dimension were lost after removing the cases with more extreme values, this suggesting that the outliers in our sample, in alternative to being a mere cause of statistical noise, represented a subgroup of postmenopausal women suffering from some degree of residual climacteric (including depressive and somatic) symptoms. In addition, when excluding the outliers from the analyses, the mismatch between subjective and objective sleep impairment in association with depressive symptoms became even less evident in postmenopausal women, when more severe affective symptoms specifically related to guilty and punishment feelings associated with both impaired subjective (BNSQ insomnia) and objective (lower sleep efficiency, shorter total sleep time and longer SWS latency) sleep quality.

It warrants attention that the significance of the associations with the depressive-somatic pattern in perimenoapusal and postmenopausal women has to be interpreted with caution. In fact, the BDI sleep-related items (#16 and #17) were found to most markedly load at the depressive-somatic factor in our model; therefore, it cannot be ruled out that these findings are again mainly expression of poor sleep in perimenopausal and postmenopausal women. However, items other than the sleep-related ones (e.g., the lost interest in sex) remarkably contributed to the depressive-somatic pattern in the factor analysis, which could in fact account for the associations detected in the perimenopausal and postmenopausal groups.

It warrants attention that all the younger women in our sample were taking OCs, and therefore, it is not possible to rule out that our results are biased by the effect of hormonal contraception. Indeed, even though a number of studies have found no difference in terms of the subjective sleep quality between naturally cycling women and OC users [73,74], a modulation of sleep architecture by OC use has been shown [73-77]. Sleep alterations, such as a reduced sleep latency [77], and specifically a short REM sleep latency [31,74,76], a decrease in SWS [73-75] but an increase in stage 2 [73] and in REM sleep [77], reduced arousals and a tendency towards a better sleep efficiency [31,78] have been reported. Burdick and colleagues [77] reported that both depressed and healthy OC users had a shorter REM latency and less SWS than non-users. Moreover, healthy OC users, but not the depressed ones, had a shorter sleep latency and a greater REM percentage than non-users [77]. On the other hand, OC use may itself be associated with adverse effects on mood in a subgroup of vulnerable women [79,80], and they are a common cause of OC discontinuation as well.

One of the limitations of this study is the small sample size. However, the numbers are in line with those of other studies on the same topic. Second, the study was carried out on a selected healthy population, thus preventing the generalization of the results to the general population. As women with previous mental disorders, such as depressive disorders, were excluded, our results cannot be extended to hold for depressed patients. In addition, the associations between sleep and depressive symptoms were limited, as only a minority of the study participants suffered from mild to moderate depressive symptoms; for this reason, it is plausible that some of our findings were in fact driven by the sleep symptoms themselves. However, given that the BDI sleep-related items loaded mostly at the somatic factor identified in our model, this limitation potentially concerns only the associations with the depressive-somatic pattern in perimenopausal and postmenopausal women. The characteristics of our study sample (a small sample, with a wide age range, that was screened for psychiatric illness and that reported few depressive symptoms) limit the applicability of the derived BDI factors to other (general and clinical) populations. Because of the recruitment criterion of three different reproductive states, the women also belonged to three different age groups. Hence, we were not able to clearly disentangle the effect of age from the effect of reproductive state on mood and sleep. However, after controlling for age within each reproductive group, part of the age effect could be taken into account. Even though we could not control for other confounding factors (e.g. stressful life events), the strict exclusion criteria allowed us to minimize the effects of factors such as irregular sleep-wake schedules, use of HT and other medications, smoking, and use of alcohol or drugs. Even though the state of mood and insomnia were assessed with a self-administered tool, nevertheless, the validity and reliability of the BDI and of the BNSQ scale has been widely demonstrated [37,81]. Furthermore, using the factor analysis procedure allowed us to better identify the associations that would have not emerged, if only the total BDI score was considered. Finally, the use of PSGs allowed us to specifically assess the objective sleep quality.

## Conclusions

Our results support the evidence of a relationship between mood and sleep, with more severe depressive symptoms being linked to sleep impairment. The notable implication of our study is that when depressive symptoms are mild, the BDI total score may not be able to detect subtle connections. In these cases, a more detailed factor analysis may offer a valuable analytical tool. Moreover, the reproductive state should be taken into account when evaluating the effect of depression on sleep, since different related depression clusters may be relevant.

## Competing interests

The authors declare that they have no competing interests.

## Authors’ contributions

SLH, NK, PP-K, ASU, TP and TP-H contributed to the conception and design of the study; NK, PP-K and ASU contributed to the acquisition of data. ET performed the statistical analyses and wrote the first draft of the manuscript. All the authors significantly contributed to the interpretation of the results and to the final draft of the manuscript, and critically revised it for its intellectual content. All authors read and approved the final manuscript.

## Pre-publication history

The pre-publication history for this paper can be accessed here:

http://www.biomedcentral.com/1471-244X/14/177/prepub

## Supplementary Material

Additional file 1 Pairwise comparison of the participants characteristics. Click here for file

Additional file 2: Figure S1 Associations between BDI factors and subjective (BNSQ insomnia) and objective sleep quality in younger women (potential outliers included). All the BDI factors resulted associated with objective sleep quality. The depressive-emotional factor (F3) especially was associated with both subjective and objective sleep quality. Note: BDI = Beck depression inventory; BNSQ = basic Nordic sleep questionnaire; REM = rapid eye movement; SWS = slow wave sleep. Click here for file

Additional file 3: Figure S2 Associations between BDI factors and subjective (BNSQ insomnia) and objective sleep quality in perimenopausal women (potential outliers included). The depressive-emotional (F3) and depressive-somatic (F4) factors were associated with subjective sleep quality; dissatisfaction (F2) and depressive-somatic symptoms (F4) were associated with objective sleep quality. Note: BDI = Beck depression inventory; BNSQ = basic Nordic sleep questionnaire; REM = rapid eye movement. Click here for file

Additional file 4: Figure S3 Associations between BDI factors and subjective (BNSQ insomnia) and objective sleep quality in postmenopausal women (potential outliers included). Punishment (F1) was associated with subjective sleep quality and the depressive-somatic factor (F4) with objective sleep quality. Note: BDI = Beck depression inventory; BNSQ = basic Nordic sleep questionnaire; REM = rapid eye movement; SWS = slow wave sleep. Click here for file
