# Supplementary material for: The relationship between mood and sleep in different female reproductive states
Source: BMC Psychiatry. 2014 Jun 16;14:177. doi: 10.1186/1471-244X-14-177 (PMC4071019; doi:10.1186/1471-244X-14-177)
Supplement: Additional file 1 — Pairwise comparison of the participants characteristics. [file 1471-244X-14-177-S1.doc]

**Additional File 1.** **Table. Pairwise comparison^ of the participants characteristics**

|  | **Younger *vs.* perimenopausal** | | **Younger *vs.* postmenopausal** | | **Perimenopausal *vs.* postmenopausal** | |
| --- | --- | --- | --- | --- | --- | --- |
|  | Mean (SD; range) | *p*-value | Mean (SD; range) | *p*-value | Mean (SD) | *p*-value |
| **Age**  (years) | 23.1 (1.6; 20-26) *vs.*  47.7 (2.3; 43-51) | *<0.001* | 23.1 (1.6) *vs.*  63.3 (3.6; 58-71) | *<0.001* | 47.7 (2.3) *vs.* 63.3 (3.6) | *<0.001* |
| **BDI score** | 1.8 (1.7; 0-5) *vs.* 4.1 (3.3; 0-11) | 0.147 | 1.8 (1.7) *vs.* 6.3 (3.9; 1-15) | *0.003* | 4.1 (3.3) *vs.* 6.3 (3.9) | 0.147 |
| **BNSQ insomnia** | 10.4 (2.3; 7-14) *vs.* 13.9 (3.8; 7-22) | *0.036* | 10.4 (2.3) *vs.* 16.2 (3.6; 5-22) | *<0.001* | 13.9 (3.8) vs. 16.2 (3.6) | 0.069 |
| **sleep latency** (min) | 14.1 (9.2; 3.4-33.5) *vs.*  15.2 (15.8; 1.9-64) | 1.773 | 14.1 (9.2) *vs.* 16.8 (14.4; 1-48.5) | 2.775 | 15.2 (15.8) *vs.* 16.8 (14.4) | 1.353 |
| **sleep efficiency** (%) | 93.4 (5.6; 78.2-98.7) *vs.*  84.3 (9.3; 70.6-88.7) | *0.027* | 93.4 (5.6) *vs.* 80.2 (11.4; 38.8-94) | *<0.001* | 84.3 (9.3) *vs.* 80.2 (11.4) | 0.804 |
| **total sleep time**  (min) | 448.2 (27.2; 375-474.5) *vs.*  404.9 (44.8; 339.3-425.9) | *0.030* | 448.2 (27.2) *vs.*  385.0 (54.9; 186.0-451.5) | *<0.001* | 404.9 (44.8) *vs.* 385.0 (54.9) | 0.804 |
| **number of arousals** | 75.8 (23.0; 45-129) *vs.*  97.2 (45.8; 31-200) | 0.600 | 75.8 (22.9) *vs.*  123.8 (70.8; 37-364) | 0.066 | 97.2 (45.7) *vs.* 123.8 (70.8) | 0.687 |
| **REM latency** (min) | 107.9 (36.3; 64.5-159) *vs.*  83.5 (31.1; 32.5-150.0) | 0.156 | 107.9 (36.3) *vs.*  74.3 (25.5; 38.0-171.0) | *0.012* | 83.5 (31.1) *vs.* 74.3 (25.5) | 0.888 |
| **REM %** | 21.0 (4.2; 13.7-29.4) *vs.*  20.6 (6.2; 12.3-32.5) | 1.689 | 21.0 (4.2) *vs.* 18.1 (5.3; 4.2-35.1) | 0.159 | 20.6 (6.1) *vs.* 18.1 (5.3) | 0.789 |
| **REM awakenings**  (number) | 0.7 (1.1; 0-3) *vs.* 4.5 (2.4; 0-9) | *<0.001* | 0.7 (1.1) *vs.* 3.4 (3.1; 0-11) | *0.015* | 4.5 (2.4) *vs.* 3.4 (3.1) | 0.420 |
| **SWS latency** (min) | 13.6 (5.8; 5.5-25.5) *vs.*  20.3 (17.3; 2.5-62.5) | 1.299 | 13.6 (5.8) *vs.* 14.8 (16.6; 1-68) | 1.119 | 20.3 (17.3) *vs.* 14.8 (16.6) | 0.192 |
| **SWS %** | 24.2 (3.4; 16.8-28.7) *vs.*  12.6 (5.8; 1.6-23.3) | *<0.001* | 24.2 (3.4) *vs.* 17.8 (7.5; 2.4-33.0) | *0.030* | 12.6 (5.8) *vs.* 17.8 (7.5) | 0.063 |
| **SWS awakenings**  (number) | 0.2 (0.4; 0-1) *vs.* 1.4 (1.5; 0-5) | *0.042* | 0.2 (0.4) *vs.* 2.4 (1.7; 0-7) | *<0.001* | 1.3 (1.5) *vs.* 2.4 (1.7) | 0.054 |
| **total SWA** (min) | 284.6 (89.9; 130.1-391.7) *vs.* 113.2 (98.9; 28.7-493) | *<0.001* | 284.6 (89.9) *vs.*  78.2 (31.6; 36.9-148.7) | *<0.001* | 118.4 (99.4) *vs.* 78.1 (31.6) | 0.684 |

^Bonferroni corrected.

Significant items are in italic.

Note: BDI = Beck depression inventory; REM = rapid eye movement; SWA = slow wave activity; SWS = slow wave sleep.
