# Supplementary material for: The relationship between mood and sleep in different female reproductive states
Source: BMC Psychiatry. 2014 Jun 16;14:177. doi: 10.1186/1471-244X-14-177 (PMC4071019; doi:10.1186/1471-244X-14-177)
Supplement: Additional file 2: Figure S1 — Associations between BDI factors and subjective (BNSQ insomnia) and objective sleep quality in younger women (potential outliers included). All the BDI factors resulted associated with objective sleep quality. The depressive-emotional factor (F3) especially was associated with both subjective and objective sleep quality. Note: BDI = Beck depression inventory; BNSQ = basic Nordic sleep questionnaire; REM = rapid eye movement; SWS = slow wave sleep. [file 1471-244X-14-177-S2.pdf]

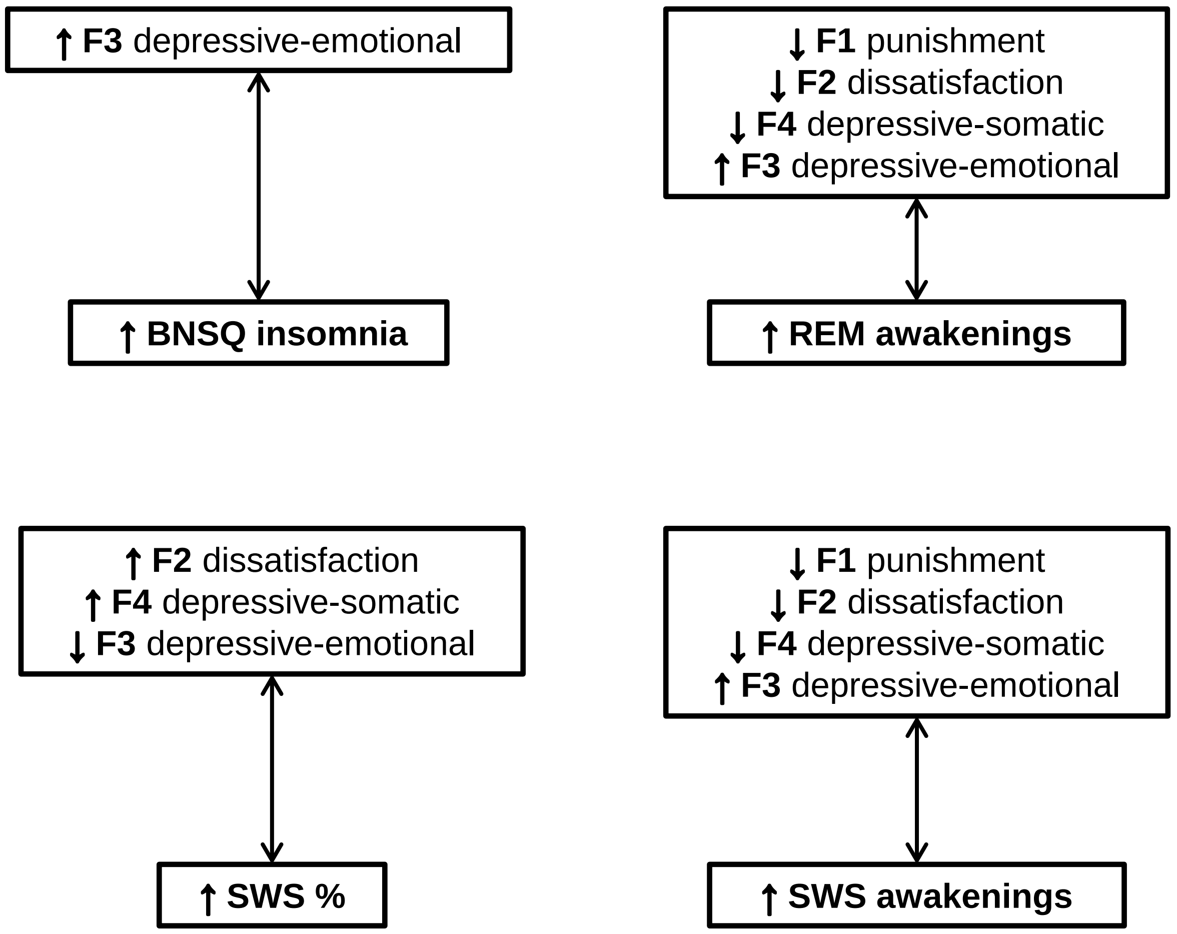

↑ F3 depressive-emotional
↑ BNSQ insomnia
↓ F1 punishment
↓ F2 dissatisfaction
↓ F4 depressive-somatic
↑ F3 depressive-emotional
↑ REM awakenings
↑ F2 dissatisfaction
↑ F4 depressive-somatic
↓ F3 depressive-emotional
↑ SWS %
↓ F1 punishment
↓ F2 dissatisfaction
↓ F4 depressive-somatic
↑ F3 depressive-emotional
↑ SWS awakenings
