# Supplementary figures and images for: The relationship between mood and sleep in different female reproductive states
Source: BMC Psychiatry. 2014 Jun 16;14:177. doi: 10.1186/1471-244X-14-177 (PMC4071019; doi:10.1186/1471-244X-14-177)

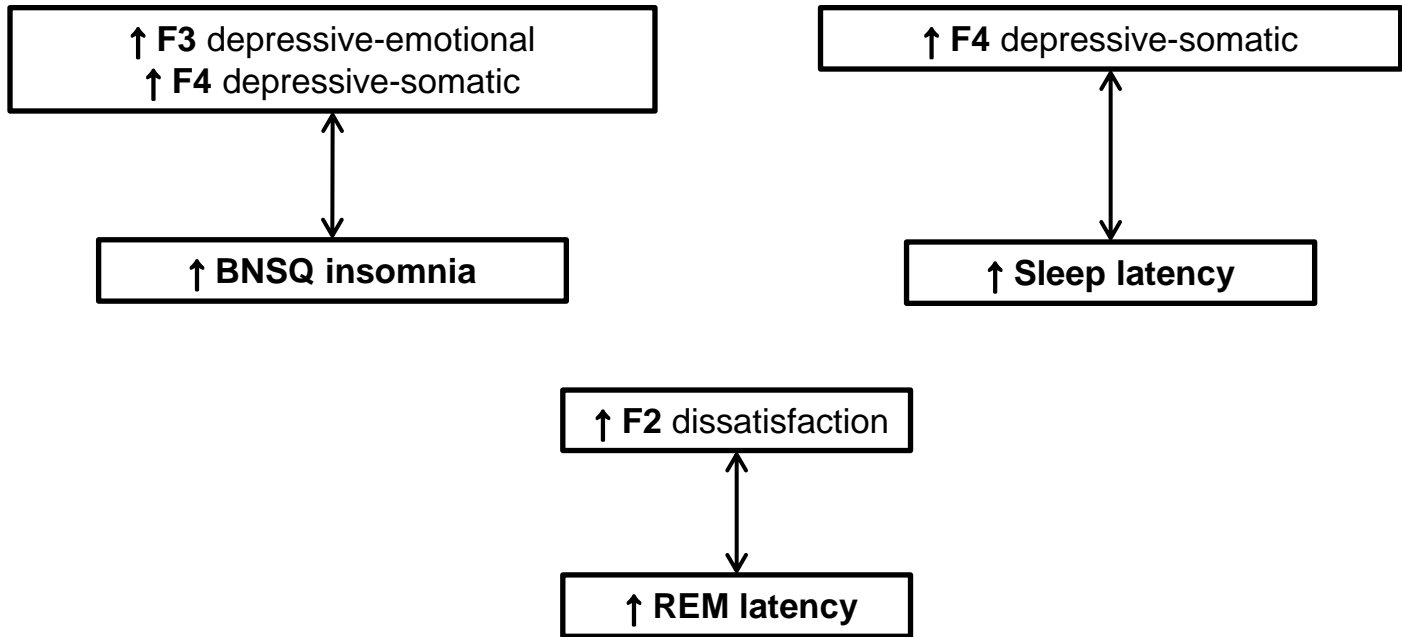

Supplement: Additional file 3: Figure S2 — Associations between BDI factors and subjective (BNSQ insomnia) and objective sleep quality in perimenopausal women (potential outliers included). The depressive-emotional (F3) and depressive-somatic (F4) factors were associated with subjective sleep quality; dissatisfaction (F2) and depressive-somatic symptoms (F4) were associated with objective sleep quality. Note: BDI = Beck depression inventory; BNSQ = basic Nordic sleep questionnaire; REM = rapid eye movement. [file 1471-244X-14-177-S3.pdf]

↑ **F1** punishment

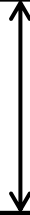

↑ **BNSQ** insomnia

↑ **F4** depressive-somatic

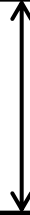

↑ **REM %**  
↑ **SWS** latency

Supplement: Additional file 4: Figure S3 — Associations between BDI factors and subjective (BNSQ insomnia) and objective sleep quality in postmenopausal women (potential outliers included). Punishment (F1) was associated with subjective sleep quality and the depressive-somatic factor (F4) with objective sleep quality. Note: BDI = Beck depression inventory; BNSQ = basic Nordic sleep questionnaire; REM = rapid eye movement; SWS = slow wave sleep. [file 1471-244X-14-177-S4.pdf]
